# Supplementary material for: Are carbon emissions trading and green financial instruments synergistic? -Comprehensive quantitative research based on content analysis
Source: PLoS One. 2024 Mar 7;19(3):e0298601. doi: 10.1371/journal.pone.0298601 (PMC10919601; doi:10.1371/journal.pone.0298601)
Supplement: S1 Data — (ZIP) [file pone.0298601.s001.zip › Data/地级市Z_F工作报告.zip!/╔╜╬≈/│ñ╓╬╩╨/└┤╫╘╣1⁄2╓┌║┼í╛┬φ┐╦╩2╛▌═°í┐_2008.docx]

各位代表：

现在，我代表市人民政府向大会作报告，请予审议，并请市政协各位委员和其他列席人员提出意见。

一、2007年政府工作回顾

2007年，在市委的正确领导下，在市人大、市政府的监督支持下，我们高举邓小平理论和"三个代表"重要思想伟大旗帜，认真学习贯彻党的十七大精神，深入贯彻落实科学发展观，大力推进工业新型化、城镇特色化、农业产业化，统筹发展社会各项事业，全市经济社会等各方面工作都取得了明显成效。

2007年，全市生产总值完成550.6亿元，比上年增长14.3％；人均生产总值达到16887元，增长13.6％；财政总收入完成125.2亿元，一般预算收入完成49.8亿元，剔除两权收入分别增长36.8％和36.5％；全社会固定资产投资完成224.5亿元，增长22％；规模以上工业增加值完成275.2亿元，增长20.8％；粮食总产量稳定在14亿公斤；社会消费品零售总额达到161.9亿元，增长21.2％；进出口总额达到1.5亿美元，增长29.3％；城镇居民人均可支配收入达到12418.5元，农民人均纯收入达到4410.5元，分别增长22.2％和13.4％；城镇登记失业率和居民消费价格总水平控制在2％和4.8％以内。在全省地区经济社会发展考核评价表彰中，我市名列第一。这里，我郑重地向大会报告，市十二届人大一次会议提出的2007年各项目标任务圆满完成！市政府向全市人民承诺的10件实事全部兑现！

2007年，主要取得了以下几方面的成绩：

（一）产业结构调整深入推进。加快传统产业新型化和新兴产业规模化步伐，重点抓了投资千万元以上的工业新型化项目260个，已建成105个项目，完成投资189亿元。传统产业素质不断提高，建成投产了潞安屯留煤矿、长钢H型钢等52个项目。新兴产业逐步壮大，突出抓了100万伏特高压、潞安集团16万吨煤基合成油等大项目的开工建设，建成投产了屯留中天10万吨甲醇、黎城聚合铝净水剂、郊区海森兽用生物疫苗等41个煤化工、装备制造、中西制药、新材料项目。旅游产业加快发展，太行山大峡谷顺利通过国家4A级景区评定验收，全市旅游总收入完成46.4亿元，增长30.3％。物流、商贸、餐饮等生产性和消费性服务业发展势头良好，完成了东明国际大酒店主体工程、紫坊物流园区、万博宜家苑建材超市等项目。

（二）城镇基础设施明显改善。全力构筑"1＋5"城市群，不断完善城市功能，各县市区建成了一批县城道路、供水供气、垃圾处理、污水处理、集中供热、绿地游园等城镇基础设施。规划建设了长治新区，建成了新区湖滨路等4条道路。加快城市重点工程建设，机场路和太长高速公路市区连接线绿化、城南生态苑、4所中小学校扩建等3项工程已完成，城隍庙广场、六府塔、气象雷达塔、客运东站、新闻中心、体育中心等6项工程主体或部分主体完工，城市热电联供、图书馆文化广场、市医院综合门诊大楼、商务大厦等4项工程开工建设。建成国省道公路205公里，村通水泥或油路率达到96.7％。

（三）社会主义新农村建设稳步推进。完善农业产业化体系，壮大了屯玉种业、长子蔬菜市场等一批生产型和流通型龙头企业，建成投资千万元以上的农业产业化项目25个；发展农户公司4800多个，从业人员达到3.5万人；培育各类农民专业合作社1394个。提高农业综合生产能力，狠抓了25个国家级农业标准化示范区建设，通过了国家标准化委员会的考核验收，成为全国第一个农业标准化示范市；实施了耕地综合生产能力建设工程，重点抓了10个高效节水园区建设，全国农田节水经验交流现场会在我市召开。改善村容村貌，突出抓了新农村的规划建设，省定132个试点村和236个重点推进村完成了规划编制工作；积极推进农村街巷硬化、村庄绿化、环境净化、道路亮化和改厕、改厨、改水、改圈，297个村达到了绿化标准，345个村达到了亮化标准，368个村达到了净化标准，发展农村沼气2.4万户。全面实施农村饮水安全工程，去年投资1.25亿元建成各类饮水安全工程382处，解决了27.1万农村人口的饮水安全问题。加大扶贫开发力度，积极推进28个省"两区"开发项目，26个项目已开工建设，其中9个项目已建成投产；继续实施移民搬迁、整村推进、培训转移、产业扶贫四大扶贫工程，全市移民搬迁4300人，培训农民10万人，转移农村剩余劳动力3.9万人。

（四）改革开放步伐加快。推进国有企业改革，对市属17户企业实施了改制，全市公交企业改制面达到73％；解决了16户破产关闭企业的遗留问题以及8000多名职工的生活问题；切实搞好企业办社会职能分离工作，第三批23所省属国有企业自办中小学全部顺利移交。放手发展民营经济，去年全市民营经济完成增加值285.3亿元，完成入库税金43.6亿元，分别增长17.8％和17.2％。大力开展招商引资工作，组织参加了第二届中博会、全省珠洽会、首届煤博会，成功举办了"山西·长治国际友好周"活动，去年共签约项目84个，其中23个项目开工建设。

（五）生态环境保护得到加强。深入开展创建国家环保模范城市活动，实施了上党盆地和长治湿地"两地"保护工程，长治湿地被建设部批准为全省首家国家城市湿地公园。加大了节能减排工作力度，全市万元生产总值综合能耗下降6.04％，二氧化硫、化学需氧量排放量在2005年的基础上分别下降6.62％和4.17％；市区空气质量二级以上天数达到313天，比上年增加28天，在全国113个监测城市中的位次由2006年第67位前移至第38位，全省城乡环境综合整治定量考核连续7年名列第一。大力推进六大造林绿化工程，完成造林面积42万亩，植树960万株，新增城市绿地面积68万平方米，全市森林覆盖率达到26.9％，城市绿化覆盖率达到45.8％。广泛开展城乡环境卫生集中整治活动，全市配备了乡镇卫生巡查员1342人，农村保洁员12017人。

（六）以改善民生为重点的和谐社会建设取得新成效。加大社会事业投入力度，去年全市财政对科技、教育、卫生、文化的投入达到24.7亿元，比上年增长28.2％。不断增强科技创新能力，实施了各类科技计划98项，获得省级以上科技进步奖13项，申请专利273件，加快了"科技信息村村通"网络建设，连续5次被评为全国科技进步先进城市。继续实施免费义务教育，为全市50万名义务教育阶段中小学生免除了学杂费，为11万名贫困家庭学生提供了免费教科书，为2.3万名贫困寄宿生发放了生活补贴；突出抓了农村中小学校建设，新建了60所寄宿制小学，完成了1750所中小学远程教育工程。着力解决群众看病难、看病贵问题，继续推进了农村卫生"四个一"工程，不断完善城市卫生支援农村卫生工作制度，建成了农村甲级卫生所2624个、花园式乡镇卫生院98个，县乡村三级医疗卫生机构达标率达到68％；新型农村合作医疗覆盖率达到100％，农民参合率达到87％；加强社区卫生服务工作，达标的社区卫生服务机构67个，覆盖率达到81％。扎实推进就业和社会保障工作，全市新增就业3.65万人，607户零就业家庭全部安排了就业；城镇职工养老、医疗、失业、工伤、生育保险覆盖面不断扩大，市属国有企业下岗职工向失业保险并轨工作顺利完成；提高了城市低保对象的补贴标准，开展了农村低保工作，5.4万名城镇低保对象和7.1万名农村低保对象实现了应保尽保；对1.3万名农村五保供养对象、3017名建国前入党的农村老党员提高了补贴和保障标准；城镇和农村大病医疗救助全面启动。全面落实人口与计生工作各项奖励扶持政策，人口自然增长率控制在5.37‰，人口预期寿命达到71.6岁。启动了"文化低保"工程，解决了835个贫困村、近30万户贫困人口的读书难、看戏难、看电影难的问题；实施文化精品工程，创作完成了《八路军》、《精卫填海》等一批优秀作品，市杂技团出访韩国进行文化交流取得成功。广泛开展群众性体育活动，竞技体育水平稳步提高，成功举办了第二届"中国长治---壶关太行山大峡谷国际攀岩精英赛"。狠抓了以道路交通、中小学校、森林防火、非煤矿山、危险化学品、易燃易爆品、公众聚集场所消防等为重点的安全专项整治，特别是加大了煤矿安全生产工作的力度，进一步完善了煤矿安全生产隐患排查、责任追究、监督奖惩等16项管理制度，集中开展了打击超层越界开采、超工作面生产、超定员下井专项整治活动，确保了煤矿安全生产形势的持续稳定好转。老龄、妇女儿童、残疾人事业健康发展，新闻出版、广播电视、外事侨务、民族宗教、文物保护、防震减灾、气象、统计、档案、保密、地方志等工作都取得了新成绩。

（七）民主法制和精神文明建设进一步加强。市政府认真执行市人大及其常委会的各项决议，积极支持市政协参政议政，主动加强与各民主党派、工商联、无党派人士和人民团体的联系，去年共办理人大代表建议176件，政协委员提案328件。广泛开展了"五五"普法和平安长治创建活动，严厉打击各种违法犯罪行为，实施了打击"黑砖窑"和公路"治超"专项行动。深入开展了社会主义荣辱观教育，大力弘扬太行精神和纪兰精神，在城乡积极推行文明和谐社区建设，精神文明建设取得了新进展。深化国防教育和"双拥共建"活动，荣获了全国双拥模范城"三连冠"。推进依法行政，强化行政监察和审计监督，党风廉政建设和反腐败工作成效明显。

各位代表，以上这些成绩的取得，是全市人民在省委、省政府和市委的正确领导下，群策群力、拼搏进取的结果。在此，我代表市人民政府，向广大工人、农民、知识分子和各级干部，向驻市人民解放军、武警部队官兵、公安干警，向各民主党派、工商联、无党派人士和人民团体，向所有关心、支持长治建设和发展的海内外各界朋友，表示衷心的感谢和崇高的敬意！

在看到成绩的同时，我们也清醒认识到，当前我市经济社会发展还存在着不少困难和问题，概括起来主要是"三个不快、三个较高、三个压力大、三个不到位"：一是产业结构还不合理，传统产业改造提升不快，新兴产业规模扩张不快，服务业发展不快。二是经济发展方式粗放，资源能源消耗较高，污染排放总量较高，资源环境代价较高。三是统筹城乡发展和加快社会建设的任务艰巨，就业和社会保障的压力大，农民持续增收的压力大，社会稳定和安全生产的压力大。四是政府自身建设有差距，社会管理和公共服务职能发挥还不到位，依法行政和科学管理还不到位，作风转变和责任落实还不到位。对于这些问题，我们要高度重视，切实采取措施加以解决，决不辜负全市人民的重托和期望。

二、2008年政府工作安排

2008年是全面贯彻落实党的十七大精神的第一年，是进一步落实省市第九次党代会工作部署、深入实施"十一五"规划的关键年，也是改革开放30周年和举办北京奥运会的喜庆年。认真做好今年的各项工作，意义深远，责任重大。

各位代表，我市正处在科学发展、和谐发展、转型跨越发展的重要时期，既面临着发展的广阔空间和难得的机遇，又面临着各种困难和挑战。综观国内外形势，经济全球化深入发展，国家不断加大对中部崛起和老工业基地振兴的支持力度，省委、省政府建设新基地新山西的战略部署全面推进，特别是近年来我市经济实力大幅提升，加快发展的内在活力显著增强，这些都为我们发展创造了有利条件。同时也要清醒地认识到，我们正处在经济社会各类矛盾的凸显期，当前经济社会发展的不确定因素增多，全球经济失衡加剧、增速放慢，国内物价上涨和通货膨胀压力增大，我市经济社会发展中一些不协调、不稳定、不可持续的问题还比较突出。因此，我们必须进一步增强大局意识、忧患意识和责任意识，努力做到更加主动地抢抓机遇，更加科学地谋划发展，更加积极地维护稳定，更加有效地破解难题，推动全市经济社会又好又快发展。

2008年政府工作的总体要求是：全面贯彻落实党的十七大精神，高举中国特色社会主义伟大旗帜，以科学发展观为统领，按照市委九届五次全会的部署，加快转变经济发展方式，不断深化经济结构调整，全力推进工业新型化、城镇特色化和农业产业化，切实改善民生，全面推动经济建设、政治建设、文化建设和社会建设，着力促进全国文明城市、国家环保模范城市、中国优秀旅游城市和宜居城市四城联创，为加快建设实力、魅力、和谐长治而努力奋斗。

2008年经济社会发展的主要预期目标是：全市生产总值增长12％；全社会固定资产投资增长24％；社会消费品零售总额增长15％；进出口总额增长10％；实际利用外资增长20％；财政总收入增长17％；一般预算收入增长19％。

以上是指导性指标，围绕优化结构、提高效益、降低消耗、保护环境，今年还要确保完成以下约束性指标：服务业增幅高于全市生产总值增幅；研发投入占全市生产总值的比例达到1％；万元生产总值综合能耗下降6.6％；二氧化硫排放量控制在13万吨以内，化学需氧量排放量控制在2.53万吨以内；居民消费价格涨幅控制在全国平均水平以下；城镇居民人均可支配收入和农民人均纯收入分别增长12％以上；城镇登记失业率控制在4％以内；人口自然增长率控制在5.5‰以内。

根据上述总体要求和主要目标，2008年市政府的主要任务是，全力打好服务业发展、循环经济、节能减排、新农村建设、城镇特色化建设"五个攻坚战"，大力实施教育均衡发展、创业就业、医疗保健、社会保障、住房安居"五大惠民工程"，切实强化改革、开放、科技、人才、安全生产"五项保障措施"，努力开创全市经济社会发展的新局面。

（一）打好服务业发展攻坚战，着力推进产业结构优化升级

发展服务业是我市产业结构优化升级的主攻方向。我们要抓紧制定出台全市服务业发展规划，建立公开、平等的服务业准入制度，设立服务业发展专项资金，落实各项扶持政策措施，力争今年服务业占全市生产总值的比重提高一个百分点，不断增强服务业对经济增长的拉动作用。

加快发展旅游业。以创建中国优秀旅游城市为目标，以太行风光、炎帝文化、革命纪念地为主题，以绿色、古色、红色为特点，以点连线、以线带片、以片扩面，打造点线片面相结合的旅游产业体系，走出依托市区、承接晋中晋城、辐射冀鲁豫的旅游业发展新路子，逐步实现由旅游资源大市向旅游经济强市的战略性跨越。以主城区为中心，不断完善旅游基础设施和配套服务功能，优化旅行社结构，各县市区都要规划建设星级宾馆，实现市区或机场到重要景点"一小时"通达，把主城区建设成集观光旅游、休闲度假、商务会展为一体的旅游中心和集散中心。积极构建三大旅游带，以太行山大峡谷、太行水乡为主建设东部峡谷风光旅游带，以八路军太行纪念馆、黄崖洞板山风景区为主建设中部红色文化旅游带，以沁源灵空山为主建设西部太岳山生态休闲旅游带。重点开发十大旅游景区，以太行山大峡谷为龙头，整合平顺天脊山等旅游资源，着力打造太行山峡谷群，探索太行山大峡谷旅游管理模式。组建旅游投资公司，拓宽旅游业发展的资本投入渠道，推进旅游业投资主体多元化。打好旅游产品宣传促销的"组合拳"，推进吃、住、行、游、购、娱六大要素的全面发展，确保全市旅游总收入比上年增长30％以上。

大力发展现代物流业。按照省委、省政府重点发展长治等六大物流园区的部署，发挥我市高速公路、铁路、航空综合立体交通优势，以现代信息技术为手段，整合改造现有商业、运输、物资、仓储等物流资源，实现传统物流向现代物流转变。建设集汽车配件、五金建材、家饰家具、农产品、小商品为一体的紫坊物流基地，重组改造和嫁接引进一批现代大型物流企业，扶持发展煤炭、焦炭、钢铁、塑钢型材等大宗货物批发市场，规划建设一批公路货运站、铁路货运站、集装箱中转站和物流配送中心，大力发展多式联运、快速客货运输、城市配送服务等现代运输方式。

统筹发展商贸、会展、中介等各类服务业。改造提升商贸餐饮等传统服务业，鼓励发展超市、连锁店、特许经营等现代经营业态，积极引进国外知名超市或大型商贸集团；加快发展技术研发、信息咨询、教育培训、商务会展、会计事务所、律师事务所等新兴服务业；大力发展社区服务、文化体育、休闲娱乐等生活性服务业；积极发展农村服务业，抓好农业生产销售、农业科技、农业信息、农村生活服务等农村社会化服务体系建设。组织召开全市服务业项目招商引资会，引导和支持全市煤炭等资源型企业投资服务业。

积极发展金融业。大力支持国有商业银行和政策性银行，做强做大长治市商业银行和长治市农村合作金融机构，力争引进1至2个股份制银行进驻长治，建立1至2个小额贷款公司，扶持发展财鑫、农源等担保公司，繁荣发展金融市场。优化金融服务，加强金融监管，支持保险、证券、财务公司等其他金融企业发展，引导各类金融机构加大对我市优势项目、优势企业、优势产业的信贷投放力度，培育5个企业上市融资。

各位代表，发展服务业既能优化产业结构，又能改善投资环境，还能扩大就业、增加地方财力。我们要把服务业放在优先发展的战略位置，在全市掀起服务业大发展的新高潮！

（二）打好循环经济发展攻坚战，着力转变经济发展方式

要把发展循环经济作为推进工业新型化、破解"因煤而立、因煤而困"历史性难题的主要途径，努力实现经济发展方式由速度型向效益型、由粗放型向集约型的转变。

全力构建新型工业体系。重点实施十大工业新型化工程，加快建设投资千万元以上的154个工业新型化项目，集中力量抓好100万伏特高压、潞安集团煤基合成油、多晶硅等标志性大项目。加快煤炭、焦炭、冶金、电力等传统产业的重组改造和转型升级，发展煤焦精深加工，扩大特种钢、优质钢产量，延伸拉长产业产品链。推进煤化工、装备制造、中西制药、新材料等新兴产业的规模扩张和集聚发展，抓好煤制甲醇、汽车制造、兆瓦级风力发电设备、矿用机械、神经生长因子、中药高效低温提取、循环镁基、塑钢型材、达利食品加工等一批科技含量高、发展潜力大、带动作用强的项目。

切实搞好循环经济试点工作。抓住我市被列为全省首批循环经济试点城市的有利时机，制定完善全市发展循环经济实施方案，编制实施煤炭、焦炭、冶金、电力、化工、农业、再生资源等重点行业领域的循环经济推进计划，抓好天脊集团、沁新公司、潞宝生态园区、城区演武社区、潞城市等循环经济试点单位和示范项目建设，努力形成以企业为主体、以市场为动力、政府大力推进、公众广泛参与的循环经济发展机制。

积极推进三个层面的循环经济发展。一是抓好企业层面的小循环，大力推行清洁生产，强化污染预防和全过程控制，搞好企业内部废渣、废水、废气、余热、余压的综合利用和监督管理，推进煤矸石制砖、煤矸石发电、粉煤灰生产建材、钢渣制造水泥以及废水的循环利用、焦炉煤气的回收利用，培育形成一批清洁型的循环经济企业；二是抓好园区层面的中循环，发挥长治高新区的辐射带动作用，推进7个市级工业园区和6个县级工业园区的生态化改造，引导区外企业向园区搬迁集中，实现园区土地集约使用、废物集中处理、热电能源共享，搞好关联企业之间的资源链、加工链和产品链的对接延伸，培育形成一批生态型的循环经济园区；三是抓好社会层面的大循环，加快市区污水处理厂配套管网建设，推进县城污水处理厂和垃圾处理中心建设，建立完善社区回收、市场汇聚、再加工利用三位一体的废旧物资回收再利用体系，广泛开展全民绿色行动，培育形成一批资源节约型的循环经济县市区。

（三）打好节能减排攻坚战，着力改善生态环境

强力推进节能降耗。采取综合性节能措施，全面推进工业、农业、交通、建筑等领域的节能工作。突出抓好全市100户重点耗能企业的节能降耗，继续实施采煤方法改革、余热余压利用等10大重点节能工程，年内要对冶金行业200立方米以下的炼铁小高炉、焦化行业碳化室高度4米以下的焦炉、电石和铁合金行业6300千伏安以下的矿热炉、建材行业直径2.2米以下的水泥磨机进行淘汰。完善市场化节能机制，组建专业节能公司。提高全社会节能意识，落实节能目标责任制，严格执行新上项目用能评估审核制度，坚决制止高投入、高耗能、高污染、低效益的项目建设。

切实抓好治污减排。抓好辛安泉水域、沁河源头、漳河南源和西源的保护，加大工业集中区的环境治理，完成9个燃煤电厂和22个焦化企业的脱硫设施建设，抓好洗煤、电石、石灰行业的清理整顿，完成主城区65个单位的燃煤锅炉改造，推进市污水处理厂的中水回用和重点用水行业的废水循环利用，加快壶关、襄垣等8个县的污水处理厂建设。按照"谁污染谁治理，谁污染谁付费"的原则，由排污企业自己治污，或交纳治污费由政府组织治理。严格落实治污减排目标责任制，认真执行环境影响评价制度，大力推行排污权交易制度，完善污染物总量减排统计、监测、考核体系，对不能完成治污减排工作任务的县市区，依法实施污染过错"项目限批"和"一票否决"。

全面实施"两地"保护工程。加大上党盆地保护力度，编制上党盆地生态环保规划，严格限制盆地内煤矿的产能，坚决制止新建扩建煤矿，切实做好采空区的塌陷治理和生态修复工作；对盆地内所有焦炭、冶金、化工、水泥等污染企业，全部安装环保设备和实施在线监测。进一步抓好长治湿地的保护工作，积极配合省政府规章立法，制定科学的湿地保护规划、修复措施和管理制度，维护湿地的自然生态。

进一步推进造林绿化工程。大力实施以身边增绿为主的六大造林绿化工程，突出抓好"1＋5"城市群生态绿化和主要交通干道沿线荒山、荒坡绿化，优选树种，科学种植，落实林权，强化管护，使造林绿化工程成为精细施工的现场、整体推进的样板、深度发展的典范，全面完成造林40万亩、植树1200多万株、通道绿化800公里、村庄绿化212个、矿企绿化152个、城市绿地46万平方米的林业生态建设任务，形成"铺天盖地、震撼人心"的绿化效果，力争我市早日进入全国一流绿化先进城市行列。

各位代表，加强节能减排，建设生态文明，关系到人民群众的生活质量和健康水平，关系到经济社会的可持续发展。我们要以"壮士一去不复还"的决心和勇气，持之以恒地抓好生态环境建设，走出一条开发与保护统一、经济发展与山水秀美共生共存的可持续发展道路，让长治天更蓝、水更清、山更绿！

（四）打好新农村建设攻坚战，着力抓好"三农"工作

认真贯彻落实党的农村政策。坚持把"三农"工作作为重中之重，全面落实中央和省一系列强农惠农政策和各项涉农补贴，不断完善财政支农资金的稳定增长机制，充分调动农民生产积极性，促进农业规模发展、农民持续增收。

不断提升农业产业化水平。采取"龙头企业＋基地＋农民专业合作社＋农户＋保险"的形式，健全风险共担、利益共享的农业产业化机制。做强做大一批年销售收入亿元以上的农业产业化龙头企业，规划建设32个农业科技示范园区，培育形成玉米良种、优质小麦、沁州黄小米、长子大青椒等12个集中连片的优势农产品标准化生产基地，重点抓好长治县金科、郊区百泉等10个标准化健康养殖示范项目。积极发展农民专业合作社和农户公司，进一步提高农民进入市场的组织化程度。加快市县两级农产品质量安全检验检测中心和市动物疫病控制中心建设，强化重大动植物疫病防控工作，保障农产品质量安全。

全面提高农业综合生产能力。加强农田水利基础设施建设，抓好全市基本农田整理和中低产田改造；继续推进六大兴水工程，加快建设沁源永和、屯留屯谷、平顺氵朋头等新水源工程，争取黎城吴家庄水库尽快立项兴建，搞好襄垣后湾、屯留屯绛等水库的除险加固。深入开展百万农民科技大培训，新发展5000个科技示范户。大力推广测土配方施肥、旱作节水农业、农业机械化等先进实用技术，切实增强农民科技致富能力。

全力抓好新农村试点村、重点推进村建设。充分发挥132个省定试点村的示范效应，以472个省定重点推进村为重点，全面推进农村"四化四改"，整体改善村容村貌。大力实施农村公益设施"六个一"工程，建设完善村级组织活动场所、文化科技活动室、村级卫生计生所、农民体育休闲场所、便民连锁超市和标准化小学，不断改善农民生产生活条件。

切实加强扶贫开发工作。积极探索以项目带产业、以创业带就业的扶贫开发新机制，加快建设28个省"两区"开发项目，确保项目尽快达产达效。突出抓好四大扶贫工程，强化机关定点扶贫，力争今年移民搬迁4000人，脱贫2万人。大力发展劳务经济，组织实施农村劳动力转移培训"阳光工程"和农民创业培训工程，启动农村"零就业"家庭培训计划，全面提高农民创业就业能力。

（五）打好城镇特色化建设攻坚战，着力推进城乡统筹发展

加快新区起步区建设步伐。全力推进起步区道路、河道、污水、供电、供水、供气等公共设施建设，着力建设一批标志性建筑，为新区全面建设奠定基础。

推进"1＋5"城市群建设。编制"1＋5"城市群发展规划，科学界定区域功能，避免重复建设，加快同城化步伐，逐步实现基础设施规划建设一体化、生态环境修复治理一体化、产业发展和布局一体化、城乡建设一体化。年内开工建设主城区环城高速公路，加快市区至长子一级公路建设，实现"1＋5"城市群20分钟通达目标。

抓好城市重点工程项目。确保六府塔、气象雷达塔、城隍庙广场、新闻中心、客运东站、体育中心主场馆、城区康园学校和市示范高中等8项工程年内完工，加快建设市医院综合门诊大楼、城市热电联供、图书馆文化广场、商务大厦、王庄小区学校等5项工程，开工建设长治企业总部、会展中心等工程。

完善县城基础设施。以创建国家和省级卫生县城、园林县城、环保模范县城为重点，继续抓好一批生活垃圾处理、集中供热供气、教育科技文化等基础设施建设项目。实施城乡环境卫生清洁工程，完善城乡环境卫生清洁长效机制，全面开展县城的绿化、美化、亮化工作。搞好小城镇规划建设，加快发展一批工业型小城镇、旅游型小城镇、商贸型小城镇，促进城乡统筹发展。

加快构建城乡公路交通网络。年内开工建设长治至安阳高速公路，抓紧长治至临汾高速公路的前期工作。加快建设太行山大峡谷晋煤外运公路改线工程，抓好208国道长治西过境段改建等一批国省道公路建设。推进村村通水泥或油路建设，完成农村公路通畅工程350公里，建设7个县城二级客运站和7个乡镇汽车站。继续加大治超力度，建立长效机制，巩固治超成果。

加强城市综合管理。加快城市管理信息化建设，推进城市数字化管理。加大城管综合执法力度，规范户外广告、招牌设置和停车场的建设管理。加强城市交通管理，保障道路有序畅通。组建市工程建设事务管理局，负责对政府投资的工程项目进行统一建设。年内开工改造4个"城中村"。完善城市土地储备制度，积极探索城市可经营项目的市场化运作，在市政建设、城市绿化等领域引入竞争机制。

各位代表，我们要从全市的整体利益、长远利益、根本利益出发，做好打基础、利长远、顺民意、得人心的事，坚持不懈地打好"五个攻坚战"，努力实现长治的转型跨越发展！

（六）大力实施"五大惠民工程"，着力保障和改善民生

坚持以人为本，切实把政府工作的重点、政策支持的重点、财力保障的重点向民生倾斜，不断满足人民群众的新期待、新要求。

围绕学有所教，推进教育均衡发展工程。促进义务教育均衡发展，健全义务教育经费保障机制，为全市城乡46万名义务教育阶段在校生免除学杂费，为全市农村36万名义务教育阶段在校生免费提供教科书，提高农村家庭经济困难寄宿生的生活费补助标准。建立完善城乡教师流动机制，继续开展城镇教师支援农村教育工作，选聘师范院校毕业生到农村任教，提高农村教师队伍的整体素质。大力发展职业教育，加强职业教育能力建设，对家庭困难以及市场急需专业的职业高中学生实行免费教育，让所有未能考取高中或大学的毕业生都能接受职业教育。提高普通高中教育质量，今年城区、郊区、潞城、襄垣、长治县等有条件的县市区率先基本普及高中阶段教育；健全高中学生资助制度，让品学兼优、家庭困难的学生顺利完成学业。重视发展高等教育、特殊教育和成人教育，努力办好人民群众满意的教育。

围绕劳有所得，推进创业就业工程。积极培育创业主体，落实创业扶持政策，设立创业奖励基金，加大创业培训力度，不断提高劳动者的创业就业能力，多渠道增加就业岗位，做好下岗职工、失地农民、大中专毕业生、退伍转业军人和残疾人的就业工作。完善面向所有困难群众的就业援助体系，落实空岗申报制度，建立零就业家庭就业帮扶长效机制。规范和协调劳动关系，建立企业职工工资正常增长和支付保障机制。加强劳动执法监察，规范劳动合同管理，执行最低工资制度和劳动保护标准，落实农民工平等就业、工资支付、社会保障等政策措施，打击非法用工行为，维护劳动者合法权益。

围绕病有所医，推进医疗保健工程。突出抓好农村三级卫生服务网络建设，重点搞好乡村医务人员能力培训，加快县乡村卫生机构达标步伐，提高城市卫生支援农村卫生工作水平，面向社会公开招聘500名"村医"，建立农村流动卫生所制度，开展新一轮农民免费健康体检工作。全面推进新型农村合作医疗制度，提高财政补助标准和筹资水平，扩大参合农民医疗受益面，建立农村特困户大病医疗二次救助体系，切实解决农村因病致贫问题。抓好城市卫生服务体系建设，强化社区卫生服务功能，推行社区卫生首诊制和家庭医生服务模式，努力实现居民小病不出社区的目标。加强卫生监督和重点传染病、地方病防治，提高医疗卫生应急处置能力。

围绕老有所养，推进社会保障工程。以非公有制企业、个体工商户和灵活就业人员为重点，扩大基本养老保险的覆盖面，推进行政和事业单位基本养老保险制度改革，建立农村养老保险制度，完善社会统筹与个人账户相结合的企业职工基本养老保险制度。加大农村五保户集中供养力度，加快农村敬老院的规划建设，力争50％的农村五保对象实现集中供养。完善城乡居民最低生活保障制度，扩大城乡低保的覆盖范围，切实做好动态管理下的应保尽保，建立低保标准与居民消费价格总水平上涨联动机制。

围绕住有所居，推进住房安居工程。以发展面向低收入家庭的保障性住房为重点，建立适应全体居民的多层次住房保障体系。抓好廉租房保障工作，实行实物配租和货币补贴相结合的办法，为城市低保住房困难家庭提供住房或住房租金补贴。抓好经济适用房建设，多渠道增加面向低收入家庭的经济适用房供给，实行"先定住户后盖房"的经济适用房建设和分配制度，帮助低收入家庭解决住房问题。抓好普通商品房建设，规范房地产市场秩序，有效抑制房价过快上涨，逐步改善群众的居住条件。

各位代表，着力解决人民群众最关心、最直接、最现实的利益问题，是各级政府的神圣职责。我们要集中力量实施好"五大惠民工程"，让人民群众共享改革发展的成果！

（七）切实强化"五项保障措施"，着力为经济社会发展提供有力支撑

继续深化各项改革。加快企业改革步伐，采取产权转让、资产重组、整体出售、依法破产等多种形式，推进工业、商贸、粮食等领域的市属国有企业改革，重点抓好长轴政策性破产清算工作；加快国有资本优化布局和战略性调整，培育形成一批具有核心竞争力的大公司大集团；认真落实国家和省、市关于发展民营经济的一系列激励扶持政策，完善中小企业信用担保体系和社会化服务体系，扶持发展一批成长型、科技型的民营企业。深化农村综合改革，稳定和完善农村土地承包关系，实行严格的土地管理制度，引导土地承包经营权有序流转；推进乡镇机构改革，启动集体林权制度改革试点，积极探索农村集体经济的实现形式和化解乡村债务的有效办法。推进财政管理体制改革，继续搞好平顺、壶关、武乡三个国家扶贫开发重点县"省直管县"财政改革试点工作，深入推进"乡财县管乡用"改革，完善部门预算、政府采购、非税收入管理等各项改革措施。

进一步扩大对外开放。拓展招商引资领域，提高招商选资水平，积极开展主题招商、定向招商和以企引企招商，争取引进世界500强和国内著名企业来我市投资发展。抓好中博会、煤博会等各类签约项目，组织参加第二届煤博会，引导企业参与大型商品交易会和博览会，实行点对点跟踪服务，切实提高合同履约率、资金到位率和项目开工率。积极筹建长治海关，努力扩大出口规模。加强与周边地区、沿海发达地区和国内外友好城市的交流合作，主动融入环渤海经济圈，不断扩大我市外向型经济的发展水平。

大力实施人才强市战略。推进公务员素质提升、高层次人才选拔培养、专业技术人才知识更新、农村乡土人才开发和优秀人才储备五大工程，吸引海外留学人员来我市创业发展，选聘1200名优秀大学生"村官"到农村基层工作。加大人才开发资金投入，畅通人才引进绿色通道，建立柔性引进人才机制，充分激发各类人才的创新激情、创业豪情和创造热情。

积极推进科技创新。加快构建以企业为主体、市场为导向、产学研相结合的技术创新体系。组织实施10项省级以上技术创新重点项目，支持潞安集团、防爆集团等企业的创新型试点，力争再培育发展1至2个省级以上重点企业技术中心。抓紧筹建北京大学·山西新能源新工业产业化中心，建设完善生产力促进中心和科技创新服务中心，继续实施"科技信息村村通"工程。全面实施知识产权战略和名牌带动战略，新增一批专利技术、驰名商标和名牌产品。

全力抓好安全生产。安全稳定、团结和谐是今年工作的重中之重。要围绕建设本质安全型城市，抓紧编制实施方案，深入开展安全隐患治理年活动，抓好煤矿、非煤矿山、道路交通、森林防火、中小学校、危险化学品、易燃易爆品、公众聚集场所等领域的安全专项整治。狠抓煤矿安全生产工作，认真落实煤矿安全生产的主体责任和政府的安全监管责任，严格执行煤矿业主（董事长）下井、驻矿安检员现场监管、重大安全隐患事前责任追究等一系列制度措施；以煤矿安全生产述职评议为切入点，全面开展瓦斯、水患、火灾、雷管炸药等安全隐患的排查治理；大力推进煤矿兼并重组工作，继续抓好煤矿安全质量标准化达标活动；严厉打击私挖滥采超层越界开采、超工作面生产、超定员下井等违法非法行为，坚决遏制各种重特大事故的发生，促进全市安全生产形势持续稳定好转。

各位代表，我们要认真抓好"五项保障措施"，切实增强工作的创造性、主动性、协调性和有效性，确保"五个攻坚战"和"五大惠民工程"取得明显成效！

（八）不断繁荣文化事业，着力提升文化软实力

大力发展文化事业。加强公益性文化基础设施建设，推进乡镇综合文化站和行政村文化大院的建设改造，大力开展群众喜闻乐见的文体活动。深入实施"文化低保"工程，推动文化信息资源共享、农村电影公益放映和农村20户以上自然村通广播电视工程。加强网络文化建设与管理，完成市区7万户有线电视数字化工程建设。

加快发展文化产业。以广播电视、新闻出版、文艺演出等优势文化资源为基础，大力发展文化旅游、文化创意、文化博览、动漫游戏、杂技表演、工艺美术等新兴文化产业，培育发展文化龙头企业，促进文化与旅游、体育、餐饮之间的融合，提高文化产业对经济发展的贡献率。大力实施文化精品战略，创作编排一批具有上党地方特色的精品力作。

推进和谐文化建设。构建社会主义核心价值体系，用社会主义荣辱观引领风尚，大力弘扬爱国主义、集体主义、社会主义思想，大力发扬太行精神和纪兰精神。切实加强社会公德、职业道德、家庭美德、个人品德和未成年人思想道德建设。全面落实创建全国文明城市的指标任务，深入开展文明单位、文明村镇、文明社区等群众性精神文明创建活动，努力提高市民的文明素质和城市的文明品位，争取我市早日进入全国文明城市行列。

（九）加强民主政治建设，着力维护社会稳定

推进民主政治建设。认真执行人大及其常委会的各项决议、决定，依法向人大及其常委会报告工作，自觉接受人大的法律监督和工作监督；积极支持人民政协参政议政，密切同各民主党派、工商联、无党派人士的联系；认真办理人大代表、政协委员的建议和提案。支持人民法院、检察院依法履行职责，支持工会、共青团、妇联等群团组织参与社会管理和公共事务。认真贯彻落实民族宗教政策，巩固团结和谐的民族宗教关系。加强基层民主建设，扩大政务公开、村务公开和厂务公开的范围和内容，完善民主管理，保障人民权益。

加强社会治安综合治理。深入开展平安创建活动，加强基层派出所、司法所和基层法庭建设，强化信访和矛盾排查调处工作，加强流动人口、出租房的服务和管理，搞好学校周边环境整治，深入开展打黑除恶专项行动，严厉打击各种违法犯罪活动。深入推进"五五"普法，搞好法律服务和法律援助工作。

进一步强化社会管理。扎实开展信用长治建设，整治和规范市场经济秩序，集中开展产品质量和食品药品安全专项整治，年内属于政府定价范围的调价项目原则上不出台。加强城市社区和农村社区建设，完善基层社会管理体制。坚持计划生育基本国策，优化人口结构，提高人口素质。完善国防动员体系，抓好国防教育、人民防空、民兵预备役工作，巩固双拥工作成果，推进军政军民共建活动。搞好第二次经济普查。关心支持老龄、妇女儿童、残疾人工作，继续做好外事侨务、防震减灾、文物保护、气象、档案、保密、地方志等各项工作。

（十）切实转变政府职能，着力提高政府公信力和执行力

坚持依法行政。各级政府及其部门都要严格遵守宪法和法律，依照法定权限和程序行使权利，履行职责，接受监督。进一步完善民主科学决策的体制机制，建立健全重大决策调查研究、集体决策、专家咨询、社会听证、决策评估和决策反馈纠偏机制，提高政府决策的民主化、科学化水平。认真贯彻《突出事件应对法》，建立完善预防和处置各种突发事件工作机制，提高政府应急管理能力。严格落实行政执法责任制，加强行政执法队伍建设，切实做到有权必有责、用权受监督、侵权要赔偿、违法要追究。

优化政务环境。深化行政审批制度改革，加强政务大厅建设，继续减少行政审批事项，精简办事程序，实行网上审批和电子政务监察，不断提高审批的质量和效率。认真落实国务院《政府信息公开条例》，健全信息发布制度，完善公开办事制度，提高政府工作的透明度，保障人民群众对政府工作的知情权、参与权、表达权和监督权。全面落实首办负责、限时办结、服务承诺、责任追究等制度，努力营造为人民服务、让人民满意的政务环境。

加强廉政建设。全面落实党风廉政建设责任制，扎实推进教育、制度、监督并重的惩治和预防腐败体系建设，逐步建立用制度管权、管事、管人的有效机制。继续加大源头预防和治理腐败力度，深入开展治理商业贿赂，不断引深政风行风评议，重点解决工程招投标、采矿权转让、土地征用、政府采购、医药购销等方面群众反映强烈的问题。进一步加强监察、审计工作，严肃查处各类违法违纪案件，依法严惩腐败分子，让权力在阳光下运行，使人民赋予的权力始终用来为人民谋利益，真正树立勤政廉洁的政府形象。

狠抓工作落实。大兴求真务实之风、调查研究之风、狠抓落实之风，建立一抓到底、督查到位、考核到人的工作落实机制。对政府确定的重点工作、重大项目、重要事项，实行季度通报、半年盘点、年终考核，确保工作有标准、事情有人办、效果有评价、好坏有奖惩，在全市形成重落实、敢落实、善落实的生动局面。

各位代表，按照省委、省政府和市委的工作部署，今年市政府在完成上述工作任务的同时，要着力为人民群众办好以下12件实事：一是市县乡新建、改扩建中小学校52所，新建农村寄宿制小学30所，国有重点煤矿采煤沉陷区新建中小学和托儿所14所，维修加固22所。二是城镇新增就业岗位3.69万个，劳动者创业就业8200人；培训新型农民12万人，转移农村富余劳动力3.5万人。三是县乡村三级医疗卫生机构基础设施75％达标；城市社区卫生服务机构建设完成规划任务的85％以上；国有重点煤矿采煤沉陷区新建医院2所、维修加固1所。四是集中治理25个地质灾害严重村，解决2175户、8818名农民的住房安全问题和严重饮水困难；城市居民棚户区改造新建住宅5万平方米，使550户、1650人改善居住条件；新建经济适用房18万平方米，解决2159户城市低收入家庭的住房困难；新建廉租住房3万平方米，让600户廉租住房保障对象住上新房；启动解决农村困难群体的住房问题。五是全年二级以上天数达到315天以上，环境空气质量稳定达到二级标准。六是新解决38万农村人口饮水安全问题。七是深入实施"万村千乡市场工程"和新农村现代流通网络工程，发展连锁农家店300家。八是在农村发展以沼气为主的太阳能、生物质能等可再生能源用户2万户。九是完成村村通水泥或油路500公里，村通水泥或油路率达到97％。十是拓宽改造延安路等城市主干道，缓解市区交通拥挤状况。十一是建立城镇居民基本医疗保险制度，解决全市42.5万城镇居民和市属国有关闭、破产、困难企业职工及退休人员的医疗保障问题。十二是对500名贫困残疾人实施康复救助，为20个社区配备残疾人康复器材，解决200户农村残疾人的住房困难。以上12件实事，是市政府向全市人民的庄严承诺，一定说到做到，办实办好，坚决兑现，向全市人民交一份满意的答卷！

各位代表，长治是华夏文明的重要发祥地，精卫填海、女娲补天、后羿射日、神农尝草等传说，展现出上党先民认识自然、改造自然的豪迈气概。长治是潞商故里，作为晋商的一支劲旅，凭借着节俭勤奋、明礼诚信、精于管理、勇于开拓的精神，走南闯北，远涉海外，享誉天下。长治是太行精神和纪兰精神的孕育之地，在抗日战争和解决战争时期，上党老区人民不怕牺牲、浴血奋战，上演了一幕幕金戈铁马、壮怀激烈的英雄史剧；在半个多世纪的社会主义建设时期，以申纪兰为杰出代表的长治人民艰苦奋斗、锐意进取，创造了无愧于历史的辉煌业绩。在新的历史时期，我们一定要继承和弘扬这些优良传统和宝贵精神，在省委、省政府和市委的坚强领导下，高举中国特色社会主义伟大旗帜，全面贯彻党的十七大精神，深入贯彻落实科学发展观，以奋发有为的精神状态，以与时俱进的发展思路，以切实有效的工作措施，全面完成今年经济社会发展的各项目标任务，努力开创全市科学发展、和谐发展的新局面，共同谱写长治人民美好生活的新篇章！
